# Quality of life among drug-resistant tuberculosis patients on treatment in SouthWest Nigeria

**DOI:** 10.4314/ahs.v24i2.9

**Published:** 2024-06

**Authors:** Janet Bamidele, Olumide Abiodun, Kolawole Sodeinde, Terkaa Bitto, Adekunle Alabi, Callistus Akinleye, Olusola Adejumo, Olusoji Daniel

**Affiliations:** 1 Olabisi Onabanjo University Teaching Hospital, Department of community medicine and Primary care; 2 Babcock University, Community Medicine; 3 Benue State University, Department of Epidemiology and Community Health; 4 Olabisi Onabanjo University, Community Medicine and Primary Care; 5 Osun State University, Department of Community Medicine; 6 Mainland Hospital Yaba, Lagos

**Keywords:** Drug-resistant tuberculosis, quality of life, Nigeria

## Abstract

**Background:**

Drug-resistant tuberculosis (DR-TB) continues to be a public health concern. Several factors, including the disease itself, affect the quality of life of DR-TB patients. This study aimed to assess the quality of life (QOL) and associated factors of drug-resistant tuberculosis patients in Nigeria.

**Methods:**

A cross-sectional study of 165 participants using an interviewer-administered 26-item World Health Organization Quality of life Brief version (WHOQOL-BREF) tool. Two questions assessed overall quality of life and general health while twenty-four questions assessed the physical, social, psychological and environmental domains of QOL. Continuous variables were summarized using means, standard deviations while association between categorical variables were analyzed using Chisquare test. Binary logistic regression model assessed the predictors of QOL with statistical significance at p<0.05

**Results:**

Mean age was 35.63 ± 11.36. The overall quality of life was 3.96±0.82. The environmental domain had the highest mean quality of life (64.9±14.6), while the physical domain had the lowest (59.2±11.2). Marital status, family size, and support from the TB programme were associated with a good QOL.

**Conclusion:**

Overall quality of life was good. Continued financial and social support for drug-resistant tuberculosis patients on treatment by the national tuberculosis control programme is recommended.

## Introduction

Drug-resistant tuberculosis poses a persistent public health threat with an estimated 465,000 cases of multi-drug-resistant tuberculosis (MDR-TB)/rifampicin-resistant tuberculosis (RR-TB) patients reported globally in 2019.^1^ MDR/RR-TB was present in 3.3% of new cases and 18% of cases of tuberculosis that had previously undergone treatment.^1^ Drug-resistant tuberculosis is a significant public health issue, particularly in developing countries where the disease is most prevalent.^2^

The longer duration of treatment for drug-resistant tuberculosis (MDR-TB/RR-TB) compared to drug-susceptible tuberculosis patients has been a significant issue in the management of these patients.^3^,^4^ Prior to May 2016, the recommended duration of treatment was for at least 20 months. In 2016, the World Health Organization reviewed the management guidelines for drug-resistant tuberculosis and recommended a shortened regimen of 9-12 months. In addition, the cost and numerous adverse reactions of these drugs have further complicated the successful management of these patients.^3^,^4^

According to the national drug resistance survey of 2012, Nigeria had a crude prevalence rate of MDR/RR-TB of 4.8% across all treatment categories. This includes a prevalence rate of 2.9% for new cases of TB and 14.3% for people who have had TB before.^5^ In 2017, WHO approximated that 4.3% of all new tuberculosis patients and 25% of previously treated cases of tuberculosis had drug resistance (MDR/RR-TB) with an incidence rate of 12 per 100,000 population.^6^ This shows a steady increase in the burden of MDR/RR-TB in the country and poses an additional challenge to the already weak system for the control of tuberculosis in the country.

One of the End TB Strategy's goals for 2035 is to treat all tuberculosis patients, including those with drug-resistant tuberculosis.^7^ Despite the laudable goal of treatment for the physical effects of the illness, MDR/RR-TB patients have several other needs such as physiological, financial, and psychological that may adversely affect their treatment outcome and the quality of their lives.

The definition of quality of life (QOL) is an individual's perception of well-being in physical, mental, and social aspects.^9^ The physical, mental, and social components of quality of life are all intertwined.^10^ The subjective perception of a patient's daily impact of a disease and its treatment on their physical, mental, and social well-being is known as their health-related quality of life.^11^ Evaluation of the health-related quality of life is therefore a crucial health outcome and a topic of interest for researchers, policymakers, and healthcare workers.^11^

Over the years, considerable attention has been focused on the prevention of transmission of tuberculosis and the treatment outcome of tuberculosis patients.^12^ The current TB control program and clinical research have neglected the patient's perspective and assessment of their well-being in favor of outcomes related to mortality and microbiologic cure.^13^ It is well known that TB is still associated with considerable social stigma and rejection by family members and close associates which may affect the quality of life of drug-resistant tuberculosis patients.

Worldwide, studies that have evaluated the quality of life of drug-resistant tuberculosis patients found that their QOL was lower than what was seen among drug-susceptible patients and the general populace.^11^,^14^-^16^ In Nigeria, studies have been conducted to assess the quality of life of drug-susceptible tuberculosis patients.^17^-^20^ However, no study was found that has examined the QOL among drug-resistant tuberculosis patients.

In order to provide patients with drug-resistant tuberculosis with the care that is more comprehensive and patient-centered, a study evaluating their quality of life is imperative. This will help to achieve better outcomes, especially in populations with high disease burdens. Therefore, the purpose of this research was to assess the quality of life among drg-resistant tuberculosis patients in southwest Nigeria and to determine its associated factors.

## Methods

### Study setting

The study was carried out in Southwest Nigeria, consisting of six states namely Ekiti, Lagos, Ogun, Ondo, Osun, and Oyo states with a population ranging from 12,550,598 in Lagos state to 3,270,798 in Ekiti state.^21^

The southwest states are mainly inhabited by Yoruba ethnic groups who are primarily agrarian. The TB programme in the states is similarly structured and headed by a State TB programme coordinator who reports to the National TB programme coordinator. All patients detected bacteriologically to be Rifampicin-resistant positive are reported to the state TB control programme. The state team then evaluates the patient and decides whether the patient will be initiated on treatment at the available drug-resistant treatment facilities within the region or initiated within the community where the patient resides. For patients whose treatment is initiated at the treatment facility, after the initiation phase, they are discharged back to their communities to continue their treatment and are linked to the nearest outpatient drug-resistant treatment clinic for follow-up.

### Study design, population, and sample size calculation

A descriptive cross-sectional study was conducted among drug-resistant tuberculosis patients in southwest Nigeria. All drug-resistant tuberculosis patients who were ≥ 15 years and bacteriologically diagnosed using MTB/RIF Assay test, Line Probe Assay, or Sputum Culture were recruited for the study. Critically ill patients were excluded from participating in the study. A sample size of 165 was calculated using the Cochrane formula.^22^

### Sampling technique and study instrument

In this study, a multistage sampling technique was used. Out of the six states in southwest Nigeria, two were chosen by random sampling for the first stage. The states selected were Oyo and Ogun State. Secondly, a list of all facilities that reported cases of MDR-TB/RR-TB in the quarter preceding the study in the selected states was gotten from the State Ministry of health. Proportion to size allocation was used to calculate the number of drug-resistant cases to be selected from each facility. Out of 15 facilities that reported at least one case of MDR-TB/RR-TB in the preceding quarter, only 12 of the facilities had cases at the time the study was carried out. In the final stage, participants were selected from the facilities. Simple random sampling was used to choose the necessary number of participants for the study in facilities where there were more eligible patients than could be accommodated. In facilities that had fewer eligible participants than required, all eligible patients were recruited for the study.

Data collection was done using a semi-structured interviewer-administered questionnaire which was adopted from the World Health Organization Quality of Life BREF (WHOQOL BREF) questionnaire.^23^ The questionnaire also had questions relating to sociodemographic characteristics. The questionnaire was pretested among 20 drug-resistant tuberculosis patients in another southwest state which shared similar sociodemographic characteristics as the selected states.

### Reliability of WHOQOL BREF Tool

The reliability of the WHOQOL BREF tool was assessed using Cronbach's alpha analysis. To assess internal consistency, Cronbach's reliability analysis was performed on the overall quality of life scale as well as on each of the four domains. Cronbach's alpha coefficient was between 0.551 and 0.893. It was highest for overall QOL (0.893) and least for the social domain (0.551).

### Measurement of variables

Quality of life was the dependent/primary outcome in this study while demographic, socio-economic variables, social support, clinical characteristics, and treatment-related factors were the independent variables. Quality of life was assessed using the 26 questions of the WHOQOL BREF. Two questions assessed the overall quality of life and satisfaction with general health. The remaining 24 questions assessed the quality of health under the following 4 domains; physical health domain, psychological health domain, social relationship domain, and the environmental domain. For each item a Likert scale that ranged from 1 if the response was “very poor/very dissatisfied/or not at all” to 5 if it was “very good/very satisfied/or extremely” was used.

The physical health domain was assessed using responses to seven items. This domain incorporated the following: activities of daily living, dependence on medicinal substances and medical aid, energy, and fatigue, mobility, pain and discomfort, sleep and rest, and work capacity. The psychological health domain was assessed using responses to six items. The domain assessed bodily image and appearance, negative feelings, positive feelings, self-esteem, spirituality/religious/personal beliefs, thinking, learning, memory, and concentration. The social relationship domain was assessed with responses to three items. The domain assessed personal relationships, social support, and sexual activities. The environment domain was assessed using responses to eight items. The domain assessed financial resources, freedom/physical safety/security, accessibility and quality of health and social care, home environment, opportunity for acquiring new information and skills, participation in and the opportunities for recreation and leisure activities, physical environment, and transport.

Domain scores were scaled in a positive direction of 1-5 with 5 being the most optimistic response.^24^ The raw score of each domain was transformed into a standardized score of 0-100 for uniformity of scores with higher scores indicating higher quality of life.^23^,^25^ However, the overall QOL and satisfaction with general health scores were not transformed and were analyzed using the raw scores (maximum = 5) as was described in previous studies for comparability of results.^26^ Categorization into “good” or “poor” was done using the mean respondents' score of 50 as the cut-off for each of the domains while mean of 2.5 was used as the cut-off for overall quality of life and satisfaction with general health.

### Statistical analyses

The questionnaires were checked and cleaned and then entered into the Statistical Package for Social Sciences (SPSS) version 20 for analysis and statistical calculations. The analyzed data was presented as tables and charts. Data were summarized using means, standard deviations, and proportions. The primary outcome variable (quality of life) was quantitative (mean ± standard deviation). Further analysis included categorization of the primary outcome variable and the Chi-square test was used to compare the association between categorical variables (such as demographics, socio-economic, clinical, and treatment-related variables) and the quality of life. Multivariate analysis was done using backward elimination logistic regression to identify factors associated with quality of life. The level of significance was set at a 95% confidence interval with a p-value < 0.05.

## Results

A total number of 165 respondents participated in this study with a 100% response rate. The sociodemographic characteristics of respondents are shown in Table 1. The highest number of respondents were in the age group 21-30 years with a mean (± standard deviation) of 35.6±11.4. Most respondents were males 67.9% and a large number of respondents were married. The highest number 82(49.7%) orespondents were found to have a secondary level of education. Only a little over half (53.9%) of the respondents were employed. About a tenth (10.3%) of the respondents were HIV positive.

**Table 1:** Socio-demographic characteristics of respondents

Variable	Drug-resistant n (%) N=165
**Age**	
≤ 20	15(9.1)
21 – 30	54(32.7)
31 – 40	41(24.8)
41 – 50	33(20.0)
> 50	22(13.4)
Mean age =	35.6±11.4
**Sex**	
Male	112(67.9)
Female	53(32.1)
**Marital status**	
Married	80(48.5)
Single	68(41.2)
Separated	12(7.3)
Widow/widower	5(3.0)
Divorced	0(0.0)
**Family type**	
Monogamous	121(73.3)
Polygamous	44(26.7)
**Family size**	
≤ 4	58(35.2)
5-6	66(40.0)
**≥ 7**	41(24.8)
Mean family size =	5.6±2.3
**Tribe**	
Yoruba	129(78.3)
Igbo	22(13.3)
Hausa	8(4.8)
Others	6(3.6)
**Religion**	
Christianity	94(57.0)
Islam	69(41.8)
Traditional religion	1(0.6)
Others	1(0.6)
**Educational Level**	
No formal education	18(10.8)
Primary	33(20.0)
Secondary	82(49.7)
Tertiary	32(19.5)
**Current employment status**	
Employed	90(54.5)
Unemployed	75(45.5)
**HIV status**	
Negative	148(89.7)
Positive	17(10.3)

Table 2 shows the mean quality of life score of respondents. The mean overall quality of life among drug-resistant tuberculosis was 4.0 ± 0.8 while the mean satisfaction with general health was 3.9 ± 0.9. The environmental domain had the greatest mean score (64.9 ± 14.6) while the physical domain had the lowest score (59.2 ± 11.2). Quality of life scores in drug-resistant tuberculosis patients categorized into good and poor are shown in Table 3. Overall, 80% of the respondents were categorized as having a good quality of life.

**Table 2:** Mean quality of life score of respondents

Domain	Drug-resistant N=165 mean ±SD
**Overall QOL**	4.0±0.8
**Satisfaction with General Health**	3.9±0.9
**Physical**	59.2±11.2
**Psychological**	64.7±11.8
**Social**	63.9±19.4
**Environment**	64.9±14.6

**Table 3:** Quality of life scores in drug-resistant tuberculosis patients categorized into good and poor

Domain	Quality of Life category	Drug-resistant N=165
Overall	Good	132(80.0)
	Poor	33(20.0)
General Health	Good	132(80.0)
	Poor	33(20.0)
Physical	Good	129(78.2)
	Poor	36(21.8)
Psychological	Good	142(86.1)
	Poor	23(13.9)
Social	Good	116(70.3)
	Poor	49(29.7)
Environmental	Good	136(82.4)

Although there was no association between age, sex, marital status, family type, tribe, or religion and overall quality of life among drug-resistant tuberculosis patients on bivariate analysis, marital status, and family size had significant associations with the good overall quality of life on multivariate analysis (Table 4).

**Table 4:** Association between socio-demographic factors and overall quality of life (QOL) among drug-resistant tuberculosis patients on treatment

Variable	Poor QOL N=33	Good QOL N=132	Adjusted Odd Ratio (95% Confidence interval
**Age**			
≤ 35	19(57.6)	73(55.3)	
>35	14(42.4)	59(44.7)	
**COR (95%CI)**	1.1(0.5-2.4)		
**Sex**			
Male	19(57.6)	93(70.5)	1
Female	14(42.4)	39(29.5)	0.5(0.2-1.2)
**COR (95%CI)**	0.8(0.2-3.4)		
**Marital status**			
Married	20(60.6)	60(45.5)	1
Others	13(39.4)	72(54.5)	0.3(0.1-0.8)*
**COR (95%CI)**	1.8(0.8-4.0)		
**Family type**			
Monogamous	22(66.7)	99(75.0)	
Polygamous	11(33.3)	33(25.0)	
**COR (95%CI)**	0.7(0.3-1.5)		
**Family size**			
≤ 6	20(60.6)	104(78.8)	1
>6	13(39.4)	28(21.2)	0.4(0.1-0.9)*
**COR (95%CI)**	0.4(0.2-0.9)		
**Tribe**			
Yoruba	26(78.8)	103(78.0)	
Others	7(21.2)	29(22.0)	
**COR (95%CI)**	1.0(0.4-2.7)		
**Religion**			
Christianity	21(63.6)	73(55.3)	1
Others	12(36.4)	59(44.7)	1.3(0.5-3.2)
**COR (95%CI)**	1.4(0.6-3.1)		
**Educational Level**			
None/primary	11(33.3)	40(30.3)	
Sec/Tertiary	22(66.7)	92(69.7)	
**COR (95%CI)**	1.2(0.5-2.6)		
**Current employment status**			
Employed	15(45.5)	75(56.8)	
Unemployed	18(54.5)	57(43.2)	
**COR (95%CI)**	0.6(0.3-1.4)		
**HIV status**			
Negative	29(87.9)	119(90.2)	
Positive	4(12.1)	13(9.8)	
**COR (95%CI)**	0.1; 0.454		
**Family income**			
≤ 30000	14(42.4)	68(51.5)	
>30000	19(57.6)	64(48.5)	
**COR (95%CI)**	0.7(0.3-1.5)		
**Spouse work status N = 80**			
Employed	18(90.0)	50(83.3)	
Unemployed	2(10.0)	10(16.7)	
**COR (95%CI)**	^+^0.7		
**Family support**			
Yes	28(84.8)	117(88.6)	
No	5(15.2)	15(11.4)	
**COR (95%CI)**	0.7(0.2-2.1)		

* Statistically significant

Although the use of illegal drugs was initially statistically significant on bivariate analysis, this was not sustained on multivariate analysis. Multivariate analysis showed that there was an association between receiving financial support from the TB programme and overall quality of life (Table 5).

**Table 5:** Association between TB programme support, substance use, clinical variables and overall quality of life (QOL) among drug-resistant tuberculosis patients on treatment

Variable	Poor QOL N=33	Good QOL N=132	Adjusted Odd Ratio (95% CI)
**TB programme support**			
Yes	19(57.6)	103(78.0)	1
No	14(42.4)	29(22.0)	0.3(0.1-0.8)*
**COR (95%CI)**	0.4(0.2-0.9)		
**Currently smoke cigarettes**			
Yes	2(6.1)	2(1.5)	1
No	31(93.9)	130(98.5)	0.0(0.0)
**COR (95%CI)**	4.2(0.6-30.9)		
**Currently drink alcohol**			
Yes	4(12.1)	5(3.8)	1
No	29(87.9)	127(96.2)	1.0(0.1-16.2)
**COR (95%CI)**	3.5(0.9-13.9)		
**Currently use illegal drugs**			
Yes	3(9.1)	0(0.0)	1
No	30(90.9)	132(100.0)	6.0E+17(0.0)
**COR (95%CI)**	5.4(3.9-7.5)		
**Sickness (weeks) before diagnosis**			
≤ 4	11(33.3)	43(32.6)	
**5 and above**	22(66.7)	89(67.4)	
**COR (95%CI)**	1.0(0.5-2.3)		
**Onset Weight(kg)**			
≤ 51.0	21(63.6)	75(56.8)	
>51.0	12(36.4)	57(43.2)	
**COR (95%CI)**	1.3(0.6-2.9)		
**Present weight**			
≤ 54	17(51.5)	57(43.2)	
55 and above	16(48.5)	75(56.8)	
**COR (95%CI)**	1.4(0.7-3.0)		
**Comorbidities present**			
Yes	9(27.3)	18(13.6)	1
No	24(72.7)	114(86.4)	2.5(0.9-7.1)
**COR (95%CI)**	2.4(0.9-5.9)		
**Daily pills**			
≤ 4	0(0.0)	0(0.0)	
5 and above	33(100.0)	132(100.0)	
**COR (95%CI)**	^+^1.0		
**Treatment Phase**			
Intensive	17(51.5)	67(50.8)	
Continuation	16(48.5)	65(49.2)	
**COR (95%CI)**	1.0(0.5-2.2)		
**Number of days on treatment**			
≤ 62.0	14(42.4)	50(37.9)	
63.0 and above	19(57.6)	82(62.1)	
**COR (95%CI)**	1.2(0.6-2.6)		
**Drug adherence**			
Low	9(27.3)	25(18.9)	
High	24(72.7)	107(81.1)	
**COR (95%CI)**	1.6(0.7-3.9)		
**Occurrence of adverse event**			
**Yes**	32(97.0)	122(92.4)	
**No**	1(3.0)	10(7.6)	
**COR (95%CI)**	2.6(0.3-21.3)		

* Statistically significant

## Discussion

This study focused on the quality of life and factors affecting the quality of life among drug-resistant tuberculosis on treatment in Southwest Nigeria. From this study, the highest mean score was 64.9 ± 14.6 in the environmental domain. This is in contrast to what was reported in other studies done among drug-resistant patients in Saudi Arabia and Mumbai which reported the highest mean scores of 67.7 ± 14.8 and 68.6 ± 21.1 respectively in the social domain.^26^,^27^ The good social support system in the latter countries may be responsible for the difference observed in our findings.ife. Having a relatively constant source of income from the national programme while being sick and unable to perform usual economic activities was a booster to the general well-being of drug-resistant respondents compared to those who did not receive any form of support at the time of this study. It should however be noted that the financial support provided for these patients was from non-governmental organizations and the Global fund. The continued support of these patients is dependent on the continued support of these bodies. The government needs to increase financial resources for the control of TB and provide support to MDR-TB/RR-TB patients in treatment.

This study has its limitations. First it was a cross-sectional study and as a result, the study is unable to give information on whether the quality of life of the patients improved or worsened over time. Further studies should be done, using a longitudinal approach, to determine whether the quality of life will improve further with the continuation of treatment. Another limitation is that the study was conducted during the COVID-19 pandemic necessitating the diversion of attention and resources from endemic programmes like tuberculosis control to the prevention and transmission of COVID-19. This may have affected the perception of the patients about their quality of life and thus may have affected the results obtained from this study.

Despite the study's limitations, it has demonstrated that drug resistant tuberculosis patients in Southwest Nigeria, generally have good quality of life with highest scores being in the environmental domain and the least scores in the physical domain The predictors of good quality of life were marital status, family size and financial support. The financial support received by these patients is mainly frothe Global Fund for AIDS TB and Malaria unlike drug susceptible tuberculosis patients who do not receive any form of financial support while undergoing treatment.^28^ It is imperative that patients continue to receive financial and social support to sustain the good quality of life. This may serve as an incentive for patients to complete their treatment and have positive outcomes. The Government of Nigeria and National Tuberculosis Program should seek for ways to take ownership of the financial support given to patients to ensure sustainability of the program and further improve the quality of life of these patients.

## Data Availability

The data underlying this article will be shared upon reasonable request to the corresponding author.
